# Oxidative stress in human facial skin observed by ultraweak photon emission imaging and its correlation with biophysical properties of skin

**DOI:** 10.1038/s41598-020-66723-1

**Published:** 2020-06-15

**Authors:** Katsuhiko Tsuchida, Masaki Kobayashi

**Affiliations:** 1 Shiseido Global Innovation Center, Yokohama, Japan; 2 Graduate Department of Electronics, Tohoku Institute of Technology, Sendai, Japan

**Keywords:** Health care, Biochemistry, Skin cancer, Imaging, Skin diseases

## Abstract

Oxidative stress is associated with skin ageing and disease in humans. However, it is difficult to evaluate the effects of oxidative stress on the skin *in vivo* using conventional invasive methods. In this study, we performed two-dimensional imaging of ultra-weak photon emission (UPE) generated by excited species in oxidative reaction to determine regional variations in oxidative stress in human facial skin and analysed the relationship between UPE intensity and biophysical properties *in vivo*. UPE imaging of the facial skin of volunteers revealed regional variations in oxidative stress. The nose, its surrounding regions, and the area between eyebrows showed higher UPE intensity than other facial regions, indicating high oxidative stress in these regions. In contrast, only the region surrounding the eyes showed age-related alterations in UPE intensity; moreover, wrinkle score in these regions was correlated with UPE intensity. These results suggest that oxidative stress in the skin induces wrinkle formation. UPE intensity was correlated with porphyrin score in the skin; however, no correlation was observed between UPE intensity and skin colour parameters. This study provides insights into the treatment of facial skin areas vulnerable to ageing and helps improve our understanding of topical skin diseases related to oxidative stress.

## Introduction

Oxidative stress can cause skin wrinkling and is known to be associated with skin diseases in humans. It has been suggested to play a role in the pathogenesis of human skin cancers^1^. Reactive oxygen species (ROS) are involved in the pathogenesis of several allergic and inflammatory skin diseases^2^. ROS can alter gene and protein function^3^ to dysregulate intracellular and extracellular homeostasis, thereby impairing skin function. The mitochondria, along with enzymatic reactions in the cell, are major source of ROS^4,5^; in addition UV radiation also induces ROS production. UV radiation can cause skin complications by increasing ROS production^6,7^, and by oxidising squalene^8,9^ and other proteins^10^. Moreover, carbonylated protein levels in the stratum corneum of the skin are correlated with skin physiological parameters^11^.

Oxidative stress in the skin is conventionally measured using methods that require labelling with various molecules^12^. These were invasive and non-direct methods to investigate skin oxidation. However, ultra-weak photon emission (UPE), also known as biophoton emission, was recently used to assess oxidative stress in the skin non-invasively and directly.

UPE is generated by living organisms^13^, including humans^14,15^. The electronically excited species responsible for UPE are formed during lipid peroxidation and ROS-induced protein and nucleic acid oxidation^16^. Therefore, UPE imaging can be used for non-invasive label-free evaluation of the oxidative stress of the skin. UPE generated by the skin is visualised under highly-sensitive, cooled, charge-coupled device (CCD) cameras^17^. An increase in UPE was demonstrated in cancer-implanted nude mice by imaging^18^. We have previously shown the importance of two-dimensional UPE imaging in assessing acute oxidative stress in the skin caused by UV radiation^19^. We demonstrated that UV-induced UPE is generated not only by the epidermis but also the dermis. Moreover, the spectrum of UV-induced UPE showed a peak in the visible range.

The face is a highly specialised component of the human body, and facial skin is exposed to various adverse environmental conditions, including UV. While several studies have been conducted on facial skin, there are only a few reports on the oxidative stress of facial skin due to the limitations of conventional methods. Considering that the human face shows site-specific differences in biophysical properties^20,21^, regional variations of oxidative stress are predicted. Regional differences of blood flow, trans-epidermal water loss, stratum corneum hydration, temperature, pH, and sebum content in the face were reported. Additionally, with ageing, wrinkles around the eyes are characteristically observed^22^.

Herein, we evaluated the oxidative stress of facial skin using UPE imaging. Moreover, age-related alterations in UPE in each facial area were examined. Additionally, we examined the relationship between UPE intensity and biophysical properties of the skin as assessed by commonly used devices. Based on these results, we have discussed the effects of oxidative stress on facial skin, particularly in skin diseases.

## Results

### Regional variations in UPE intensity of facial skin

**Figure 1:**
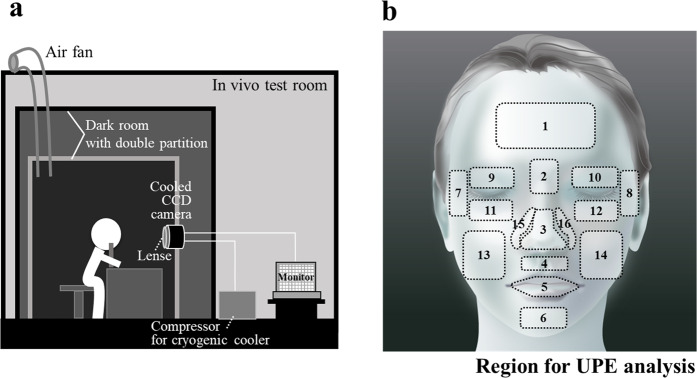
UPE imaging of facial skin and UPE analysis sites. (**a**) Volunteers were rested in a dark room with double partition. UPE images of facial skin were captured using the cooled CCD camera for 15 min. (**b**) Facial illustration of the regions used for UPE analysis. Sixteen facial areas were defined and the UPE intensity for each area was calculated from the UPE images. 1: Forehead, 2: Area between the eyebrows, 3: Nose, 4: Area under the nose, 5: Lip, 6: Chin, 7–8: Corners of eyes, 9–10: Upper eyelids, 11–12: Lower eyelids, 13–14: Cheeks, 15–16: Area around the nose.

**Figure 2:**
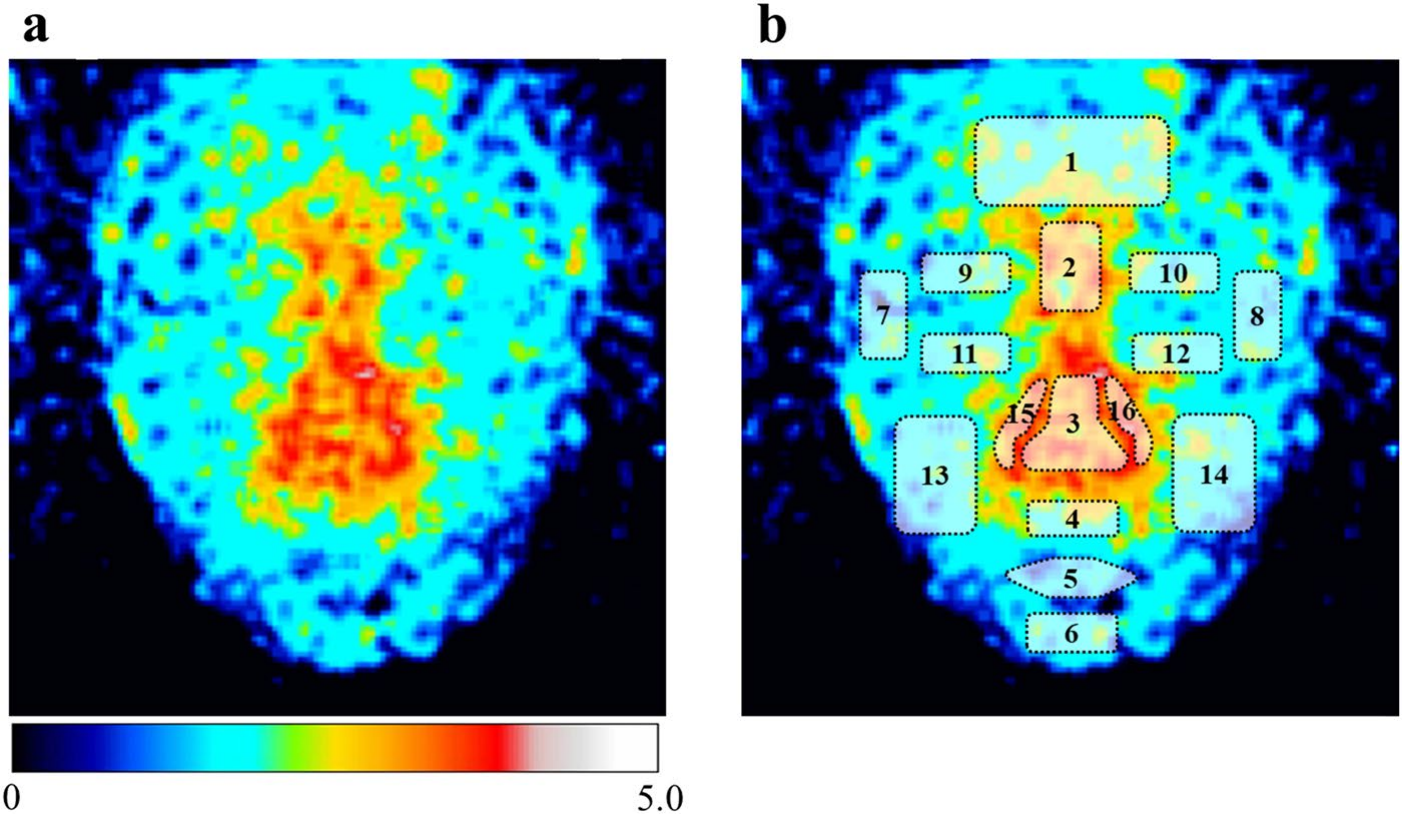
UPE image of facial skin. (**a**) Representative UPE image of facial skin. UPE imaging data were captured using a CCD camera with a 15-min exposure. The colour scale indicates signal intensity from 0 (black) to 5.0 (white). (**b**) Merged UPE image of facial skin with the regions used for UPE analysis.

**Figure 3:**
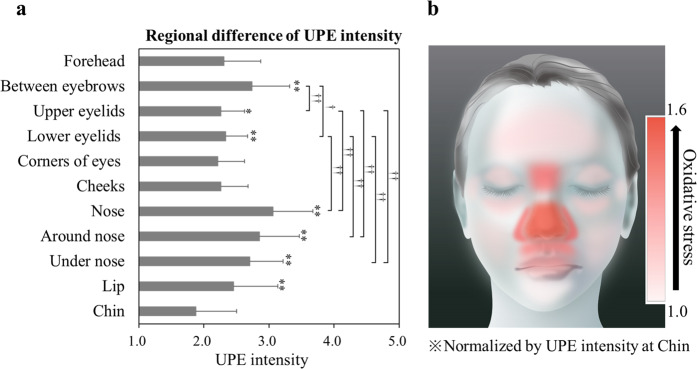
Regional variations of UPE intensity in facial skin and mapping of oxidative stress. (**a**) UPE intensity of each site was averaged for volunteers of all ages (22–69 years,) and UPE intensities of the same sites on the left and right sides were averaged. Data are presented as means ± SD, and the number of data of each site were as follows: Forehead (n = 50); area between the eyebrows (n = 50); nose (n = 50); area under the nose (n = 45), lip (n = 40); chin (n = 46); corners of eyes (n = 50); upper eyelids (n = 49); lower eyelids (n = 49), cheeks (n = 49); area around the nose (n = 50). ^*^P < 0.05, ^**^P < 0.01 vs. UPE intensity of the chin and ^†^*P* < 0.05, ^††^*P* < 0.01 (Steel-Dwass test). (**b**) Facial illustration showing the mapping of oxidative stress level normalised by UPE intensity of the chin.

Photon emission from facial skin was captured in a dark room with double partition (Fig. 1a). UPE image of the facial skin is shown in Fig. 2. Regional variations in the UPE intensity were observed for different facial areas. As shown in Fig. 1b, 16 facial areas were defined, and the UPE intensity of each area was calculated. Figure 3a shows the mean UPE intensity in each area for all volunteers. The intensities of area between the eyebrows, eyelids, nose, around and under the nose and lip were significantly higher than that of the chin. The area between the eyebrows, the nose and around and under the nose showed significantly higher UPE intensity. Figure 3b shows the mapping of the UPE intensity normalised to that of the chin with the lowest intensity for relative comparisons.

### Age-related variations in UPE intensity of facial skin

**Figure 4:**
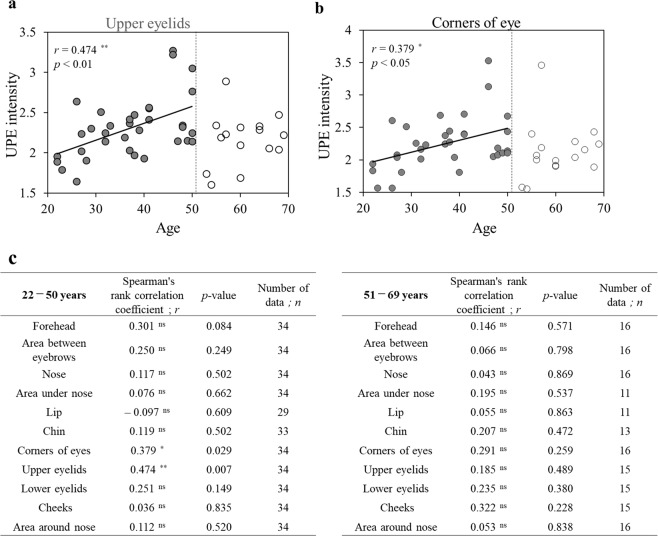
Spearman’ rank correlation test between age and UPE intensity in facial skin. (**a**,**b**) A correlation was observed between age and UPE intensity for upper eyelids or corners of the eyes in 22-to-50-year-old volunteers (closed circles) but there was no correlation in 51-to-69-year-old volunteers (open circles). (**c**) Spearman’ rank correlation coefficient and *p*-value between age and UPE intensity of each site in the face in 22-to-50-year-old and in 51-to-69-year-old volunteers. ns: not significant, *p < 0.05, **p < 0.01 (Spearman’ rank correlation test).

As shown in Fig. 4a,b, UPE intensities of the upper eyelids and areas around the corners of the eyes increased with age in 22-to-50-year-old volunteers. Furthermore, there were no age-related variations in the UPE intensity in 51-to-69-year-old volunteers.

Figure 4c shows Spearman’ rank correlation coefficient and *p*-value of each site in the face in the 22-to-50-year-old and in 51-to-69-year-old volunteers (Fig. 4c). No significant age-related changes in UPE were observed in the sites other than the upper eyelids and areas around the corners of the eyes.

### Correlation between UPE intensity and biophysical properties

**Figure 5:**
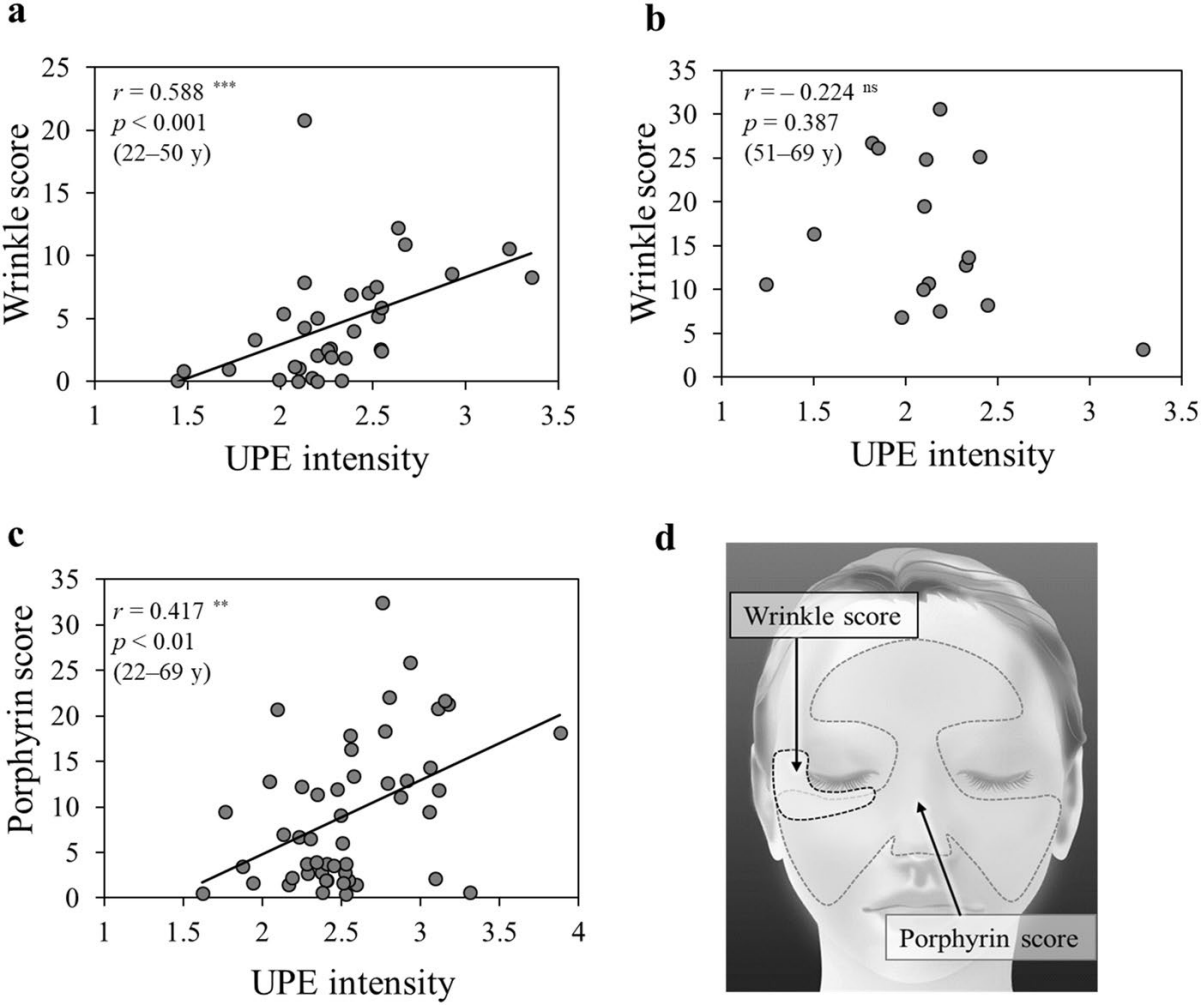
Spearman’ rank correlation test between UPE intensity and biophysical properties. (**a**,**b**) UPE intensity correlated with wrinkle score around the right eyes in 22-to-50-year-old volunteers (n = 34) but there was no correlation in 51-to-69-year-old volunteers (n = 16). (**c**) UPE intensity correlated with the facial porphyrin score for volunteers of all ages (22–69 years, n = 50). (**d**) Facial illustration indicating measurement areas for wrinkle score and porphyrin score assessment. UPE intensity corresponding to measurement areas was averaged. ns: not significant, **p < 0.01, ***p < 0.001 (S*p*earman’ rank correlation test).

**Figure 6:**
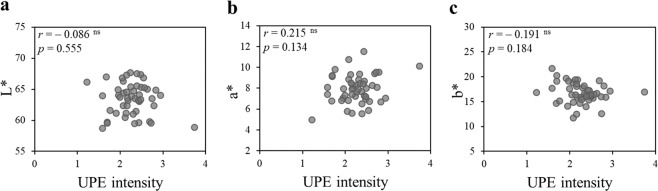
Pearson’s correlation test between UPE intensity and skin colour parameters. There was no correlation between UPE intensity of the cheek (22–69 years, n = 49) and skin colour parameters [(**a**) L* value, (**b**) a* value, (**c**) b* value]. ns: not significant (Pearson’s correlation test).

Figure 5 shows the relationship between UPE intensity and biophysical properties (e.g., wrinkling of skin, porphyrin). Wrinkle score around the right eyes significantly correlated with UPE intensity in 22-to-50-year-old volunteers (Fig. 5a). However, there was no correlation between the above-mentioned parameters in 51-to-69-year-old volunteers (Fig. 5b). Whereas porphyrin score was significantly correlated with UPE intensity in volunteers of all ages (Fig. 5c), skin colour parameters were not (L*, a*, b*; Fig. 6).

## Discussion

UPE imaging revealed regional variations of oxidative stress in human facial skin. Thereafter, the relationship between UPE intensity and biophysical properties of facial skin was investigated. Site-specific oxidation of facial skin may help elucidate the causes of various skin conditions and diseases. Moreover, site-specific oxidation of certain areas of facial skin is possible due to oxidative stress caused by high levels of sebum^23,24^ and increased susceptibility to oxidative stress by exposure to UV radiation. Regional variations of antioxidant function in facial skin are also suggested to contribute to site-specific oxidation of facial skin.

In this study, we examined chronic oxidation and its relationship with biophysical properties of facial skin. Vulnerability of the skin to oxidation could induce skin problems such as acne, rosacea, inflammation, and wrinkling. Our results showed a positive correlation between skin wrinkling and UPE intensity. Several studies have reported the high incidence of skin cancer of the nose in the face^25,26^, and our results have shown high oxidative stress in the nose. Furthermore, the role of oxidative stress in malignant melanoma and non-melanoma skin cancer^1^, and oxidative stress-associated carcinogenesis has been reported^27,28^. Increased oxidative stress can result in a higher incidence of skin cancer; hence, UPE imaging may be used for the prognosis of skin cancer.

Age-related variations in UPE intensity were observed only in the skin areas surrounding the eyes. The skin surrounding the eyes is thinner than that in other regions^29^, and experiences mechanical stress during facial movements and cosmetic application/removal during everyday life. This site is exposed to ultraviolet rays in the sunlight. Therefore, we propose that these functions with sun exposure cause age-related variations in UPE intensity and the oxidation induces wrinkling of the skin surrounding the eyes. It has been shown that ROS induces decrease and degradation of extracellular matrix (ECM) which is associated with wrinkling^30^. Additionally, previous reports established the relationship between skin wrinkling and oxidative stress using conventional methods such as surface shape evaluations and histological analyses^31,32^. Moreover, in contrast with conventional indirect methods, our method of using UPE allows direct evaluation of oxidative stress. We have previously shown that UV radiation induced UPE is generated not only in the epidermis, but also in the dermis^19^. Also, it has been reported that ROS formation during skin metabolic processes contributes to the spontaneous UPE from the skin^33^. Further, UPE derives from electronically excited species formed by ROS-induced lipid peroxidation, and protein and nucleic acid oxidation^16^. Considering the oxidative stress of the epidermis and dermis, it is reasonable to expect a correlation between skin wrinkling and UPE intensity.

UPE is also generated from UV-induced photosensitisation reaction with photosensitisers such as porphyrins. This may explain the correlation between UPE intensity and score of porphyrins excreted by bacteria. The conventional method of detecting porphyrins requires the use of a Wood’s lamp that emits long-wave UV radiation^34^. However, UPE detection may be useful to determine the levels of porphyrins without using UV radiation indirectly. Furthermore, porphyrin-induced oxidative stress is thought to be the major mechanism of porphyrin-mediated tissue damage^35^. Therefore, UPE-based detection methods can improve our understanding of porphyrin-related disorders. Finally, the a* value, which indicates skin redness, did not correlate with UPE intensity, although its relationship had been speculated because skin redness related to blood flow is affected by inflammation, which is related to the generation of oxidative stress^36^. This uncorrelation is most probably due to the absorption of visible light by blood^37^. It is therefore vital to examine the contribution of skin components, including blood, to UPE generation to further understand the underlying mechanisms. Additionally, the association between UPE and skin colour warrants further research since only the colour of cheek was measured in this study.

In conclusion, UPE imaging of facial skin revealed regional variations of oxidative stress and site-specific increases in oxidative stress with age. Moreover, a correlation was found between oxidative stress and biophysical properties of the skin. In particular, it is suggested that wrinkles formation on the face area are influence by oxidative stress. This study provides insights into the treatment of facial skin areas vulnerable to aging and helps improve our understanding of skin diseases.

## Methods

### Volunteers

Fifty healthy volunteers (Asian, female, aged 22–69 years) were enrolled in this study. The study was approved by the Ethics Committees of Shiseido Co. Ltd., and Tohoku Institute of Technology, all methods were carried out in accordance with the relevant guidelines and regulations, and written informed consent was obtained from all volunteers. The exclusion criteria for volunteers were severe atopy, allergies, sunburn, topical medication (except cosmetics), and other skin abnormalities in the face.

### UPE imaging system

A highly sensitive, cooled, CCD camera (600 series, Spectral Instruments Inc., AZ, USA) coupled with a specially designed high-throughput lens system was used for imaging. A back-illuminated CCD (original pixel format: 2048 × 2048, pixel size: 13.5 × 13.5 mm; CCD42-40, Teledyne e2v, UK) and a closed-cycle mechanical cryogenic cooler were incorporated in the camera system. The dark current of the CCD was 0.65 electron/pixel/h at −120 °C cooling and the readout noise was 4.5 electron rms. The lens system with a 0.5 numerical aperture performance was specially designed to maximise the light collection efficiency for UPE imaging. The lens magnification was 1/7, which corresponded to the imaging area of 200 × 200 mm. The total light collection efficiency to the surface of the subject was estimated to be 4.0 × 10^−3^. The CCD camera was operated in the 16 × 16 binning mode, with the actual pixel number of 128×128 (spatial resolution: 1.6 × 1.6 mm). Considering the signal-to-noise ratio of a single binned pixel, the minimum detectable number of photons for imaging was estimated to be approximately 110 photon/s/cm^2^ or 3.7 × 10^−17^ W/cm^2^ on the subject surface at the wavelength of 600 nm.

Figure 1a shows the construction of the UPE imaging system for facial skin. The imaging system consisted of a dark room with double partitions, cooled CCD camera with lens, and head-chin rest for fixing the face. A compressor for cryogenic cooler and a monitor were set outside of the dark room. An air fan was installed to prevent changes in the temperature and humidity in the dark room. All measurements were performed at 15 min exposure time.

### Measurement procedure

After washing the face with a facial cleanser to remove sebum from the surface affected by the external environment prior to measurement, volunteers wore black cape from the neck down to prevent the effects of luminescence from their clothes. Volunteers were rested in the dark room for 15 min to prevent the effect of delayed luminescence caused by external light. Spontaneous UPE images of facial skin were captured for 15 min. Wrinkle scores and porphyrin scores in the face were analysed using the VISIA system (VISIA Evolution, Canfield Scientific, NJ, USA)^38^ which analyses biophysical properties of skin from colour or UV photographs. Photographs of from the right and front sides of the face were taken. The photographs of right side were used for wrinkle analysis, while the ones of the front were used for porphyrin analysis. Wrinkle scores and porphyrin scores were calculated using factors such as the count, degree, and measurement area. Skin colour parameters (L*, a*, b*) of the cheek were measured using the CM-700d spectrophotometer (Konica Minolta, Tokyo, Japan).

### Image analysis

ImageJ software (National Institutes of Health, Bethesda, MD, USA) was used to analyse UPE images and measure UPE intensity. To remove noise from the images and blur them, the following features from the ImageJ software were applied: I. “Despeckle” and II. “Smooth”. The parts of black cape from the neck down were selected as background, the background intensity was subtracted to calculate the actual UPE intensity of the skin. UPE intensities of the 16 defined facial regions (Fig. 1b) were analysed and the UPE intensities of the same areas on the left and right sides were averaged. Sites that showed abnormal luminescence were excluded from analysis. Therefore, the number of valid data for each part of the face was as follows: 1: Forehead (n = 50), 2: Area between the eyebrows (n = 50), 3: Nose (n = 50), 4: Area under the nose (n = 45), 5: Lip (n = 40), 6: Chin (n = 46), 7–8: Corners of eyes (n = 50), 9–10: Upper eyelids (n = 49), 11–12: Lower eyelids (n = 49), 13–14: Cheeks (n = 49), 15–16: Area around the nose (n = 50). To analyse the correlation between UPE intensity and biophysical properties, UPE intensities corresponding to measurement areas for evaluation of biophysical properties were averaged.

### Statistical analysis

The Steel-Dwass test was used to verify the regional variations of UPE intensity in facial skin. The correlation between age or biophysical properties parameters, other than skin colour and UPE intensity, was evaluated using Spearman’s rank correlation coefficient. The correlation between skin colour parameters and UPE intensity was evaluated using Pearson’s correlation coefficient.

## Data Availability

The datasets generated and/or analysed during this study have not been made publicly available due to the inclusion of personal information. They may be made available by the corresponding author upon reasonable request.

## References

[1] Sander CS, Hamm F, Elsner P, Thiele JJ (2003). Oxidative stress in malignant melanoma and non-melanoma skin cancer. Br. J. Dermatol..

[2] Okayama Y (2005). Oxidative stress in allergic and inflammatory skin diseases. Curr. Drug Targets Inflamm. Allergy.

[3] Rittie L, Fisher GJ (2002). UV-light-induced signal cascades and skin aging. Ageing Res. Rev..

[4] Sabharwal SS, Schumacker PT (2014). Mitochondrial ROS in cancer: initiators, amplifiers or an Achilles’ heel?. Nat Rev Cancer..

[5] Li JM, Shah AM (2003). ROS generation by nonphagocytic NADPH oxidase: potential relevance in diabetic nephropathy. J Am Soc Nephrol..

[6] Bickers DR, Athar M (2006). Oxidative stress in the pathogenesis of skin disease. J. Invest. Dermatol..

[7] Svobodova A, Walterova D, Vostalova J (2006). Ultraviolet light induced alteration to the skin. Biomed. Pap. Med. Fac. Univ. Palacky Olomouc Czech Repub..

[8] Ekanayake-Mudiyanselage S, Hamburger M, Elsner P, Thiele JJ (2003). Ultraviolet A induces generation of squalene monohydroperoxide isomers in human sebum and skin surface lipids *in vitro* and *in vivo*. J. Invest. Dermatol..

[9] Uchino T, Tokunaga H, Onodera H, Ando M (2002). Effect of squalene monohydroperoxide on cytotoxicity and cytokine release in a three-dimensional human skin model and human epidermal keratinocytes. Biol. Pharm. Bull..

[10] Sander CS (2002). Photoaging is associated with protein oxidation in human skin *in vivo*. J. Invest. Dermatol..

[11] Fujita H, Hirao T, Takahashi M (2007). A simple and non-invasive visualization for assessment of carbonylated protein in the stratum corneum. Skin Res. Technol..

[12] Genov M (2016). Tetrahydroanthraquinone Derivative (±)-4-Deoxyaustrocortilutein Induces Cell Cycle Arrest and Apoptosis in Melanoma Cells via Upregulation of p21 and p53 and Downregulation of NF-kappaB. J Cancer..

[13] Havaux M, Triantaphylides C, Genty B (2006). Autoluminescence imaging: a non-invasive tool for mapping oxidative stress. Trends Plant Sci..

[14] Prasad A, Pospíšil P (2013). Towards the two-dimensional imaging of spontaneous ultra-weak photon emission from microbial, plant and animal cells. Sci. Rep..

[15] Nakamura K, Hiramatsu M (2005). Ultra-weak photon emission from human hand: influence of temperature and oxygen concentration on emission. J. Photochem. Photobiol. B Biol..

[16] Pospíšil P, Prasad A, Rác M (2014). Role of reactive oxygen species in ultra-weak photon emission in biological systems. J. Photochem. Photobiol. B Biol..

[17] Kobayashi M (2014). Highly sensitive imaging for ultra-weak photon emission from living organisms. J. Photochem. Photobiol. B Biol..

[18] Takeda M (2004). Biophoton detection as a novel technique for cancer imaging. Cancer Sci..

[19] Tsuchida K, Iwasa T, Kobayashi M (2019). Imaging of ultraweak photon emission for evaluating the oxidative stress of human skin. J. Photochem. Photobiol. B.

[20] Wa CV, Maibach HI (2010). Mapping the human face: biophysical properties. Skin Res. Technol..

[21] Marrakchi S, Maibach HI (2007). Biophysical parameters of skin: map of human face, regional, and age-related differences. Contact Derm..

[22] Akiba S (1999). Influence of chronic UV exposure and lifestyle on facial skin photo-aging–results from a pilot study. J Epidemiol..

[23] Kim BR, Chun MY, Kim SA, Youn SW (2015). Sebum secretion of the trunk and the development of truncal acne in women: Do truncal acne and sebum affect each other?. Dermatology.

[24] Lopez S (2000). Transepidermal water loss, temperature and sebum levels on women’s facial skin follow characteristic patterns. Skin Res. Technol..

[25] Vuyk HD, Lohuis PJ (2001). Mohs micrographic surgery for facial skin cancer. Clin. Otolaryngol. Allied Sci..

[26] Buettner PG, Raasch BA (1998). Incidence rates of skin cancer in Townsville, Australia. Int. J. Cancer.

[27] Sander CS, Chang H, Hamm F, Elsner P, Thiele JJ (2004). Role of oxidative stress and the antioxidant network in cutaneous carcinogenesis. Int. J. Dermatol..

[28] Toyokuni S (2006). Novel aspects of oxidative stress-associated carcinogenesis. Antioxid. Redox Signal..

[29] Chopra K (2015). A comprehensive examination of topographic thickness of skin in the human face. Aesthet. Surg. J..

[30] Kammeyer A, Luiten RM (2015). Oxidation events and skin aging. Ageing Res Rev..

[31] Shin MH, Seo JE, Kim YK, Kim KH, Chung JH (2012). Chronic heat treatment causes skin wrinkle formation and oxidative damage in hairless mice. Mech. Ageing Dev..

[32] Chiba K, Sone T, Kawakami K, Onoue M (1999). Skin roughness and wrinkle formation induced by repeated application of squalene-monohydroperoxide to the hairless mouse. Exp. Dermatol..

[33] Rastogi A, Pospísil P (2011). Spontaneous ultraweak photon emission imaging of oxidative metabolic processes in human skin: effect of molecular oxygen and antioxidant defense system. J Biomed Opt..

[34] Asawanonda P, Taylor CR (1999). Wood’s light in dermatology. Int. J. Dermatol..

[35] Maitra D (2019). Porphyrin-induced protein oxidation and aggregation as a mechanism of porphyria-associated cell injury. Cell Mol. Gastroenterol. Hepatol..

[36] Nishigori C, Hattori Y, Arima Y, Miyachi Y (2003). Photoaging and oxidative stress. Exp. Dermatol..

[37] Patterson MS, Wilson BC, Wyman DR (1991). The propagation of optical radiation in tissue. II: Optical properties of tissues and resulting fluence distributions. Lasers Med. Sci..

[38] Yin Y, Li J, Li Q, Zhang A, Jin P (2019). Autologous fat graft assisted by stromal vascular fraction improves facial skin quality: A randomized controlled trial. J Plast Reconstr Aesthet Surg..

